# The Role of the Monocyte-to-Lymphocyte Ratio in Acute Ischemic Stroke Patients with Acute Kidney Injury

**DOI:** 10.1155/2022/7911033

**Published:** 2022-08-28

**Authors:** Fen Jiang, Zhen Shi, Xiangyang Liu, Jiaxuan Xiang, Jie Lei, Bo Yang, XiangLi Lei, Xuewei Li

**Affiliations:** ^1^The First Affiliated Hospital, Department of Nephrology, Hengyang Medical School, University of South China, Hengyang, Hunan 421001, China; ^2^Division of Nephrology, Huayuan People's Hospital, Xiangxi, Hunan 416400, China; ^3^Department of Clinical Medicine, Xiangnan University, Chenzhou, 423000 Hunan Province, China; ^4^Affiliated Nanhua Hospital, Department of Nephrology, Hengyang Medical School, University of South China, No. 336 Dongfeng Road, Hengyang, Hunan 421001, China; ^5^The First Affiliated Hospital, Department of Neurology, Hengyang Medical School, University of South China, Hengyang, Hunan 421001, China

## Abstract

**Objective:**

The objective of the study was to determine if acute kidney injury (AKI) in patients with acute ischemic stroke was associated with the monocyte-to-lymphocyte ratio (MLR) assessed upon admission to the neurology intensive care unit (NICU) (AIS). We also looked into the MLR's function in predicting hospital mortality in AIS patients.

**Methods:**

This retrospective analysis included 595 adult patients with AIS who were hospitalized to the NICU of the First Affiliated Hospital of South China between January 2017 and December 2019. Clinical signs and imaging studies were used to diagnose AIS. KDIGO criteria were used to define AKI. The ratio of monocytes to lymphocytes was used to compute MLR, the ratio of neutrophils to lymphocytes was used to calculate NLR, and the ratio of platelets to lymphocytes was used to calculate PLR.

**Result:**

361 males and 234 women between the ages of 66.27 ± 12.05 years took part in the study. The individuals' MLR was 0.4729 ± 0.3461 and their neutrophil-to-lymphocyte ratio (NLR) was 8.18 ± 5.45. There were notable disparities in MLR and NLR between the AKI and non-AKI groups (*p* < 0.001). The link between MLR and AKI development risk was enhanced after adjustment, with respective cutoff values of 0.4581 and 9.26. For the MLR-based prediction of AKI incidence, the areas under the receiver-operating characteristic curves (AUCs) were 0.711 (95% CI: 0.663-0.758). And NLR-based prediction of AKI incidence the AUCs was (95% CI: 0.742-0826). Additionally, MLR was associated with a higher rate of in-hospital mortality (2.825, 95% confidence interval: 1.058, 7.545), whereas NLR was associated with a risk of in-hospital mortality of 1.085. (95 percent CI: 1.022, 1.151). An AUC of 0.745 (95% CI: 0.601-0.889, *p* = 0.026) was obtained for in-hospital mortality based on the MLR, whereas an AUC of 0.724 (95% CI: 0.531-0.916, *p* = 0.042) was obtained for in-hospital mortality based on the NLR.

**Conclusion:**

MLR and neutrophil-to-lymphocyte ratio (NLR) were associated with a higher risk of AKI and in-hospital death in AIS patients.

## 1. Introduction

The leading cause of mortality and disability in the world, acute ischemic stroke (AIS), has a large economic impact [1, 2]. Stroke is frequently accompanied by a number of side effects, including thrombosis, infection, and starvation. Acute kidney damage (AKI) following stroke has received more attention in recent years. It has been noted that the incidence of AKI following a stroke might range from 2.2 to 28.4 percent [3–6]. AKI also raises the likelihood of severe disability and in-hospital mortality after a stroke. Early detection and early development of AKI have important practical significance, which may lead to better clinical outcomes and reduce financial burdens. Currently, changes in serum creation (Scr) levels and urine volume are used to diagnose AKI. But previous studies have suggested that Scr is changed by many nonrenal factors and only increases after a substantial portion of nephrons has already been injured [7, 8]. Although new markers like kidney injury molecule-1 (KIM-1) and cystatin C (Cyst C) have been developed in numerous studies to help diagnose AKI early on, few have been used in clinical settings [9–11].

Inflammation has a profound impact on the development of AIS. After the stroke, a robust inflammatory response is triggered [12, 13], neutrophils and monocyte are upregulated, and lymphocytes are reduced, which are immediately induced in the peripheral blood [14]. In the meantime, AKI development involves inflammation heavily. AKI patients exhibit changes in the morphology or/and functionality of their tubular epithelium and/or vascular endothelial cells [15, 16].

A new systemic inflammatory response biomarker called the monocyte-to-lymphocyte ratio (MLR) is obtained by dividing the frequency of monocytes by the number of lymphocytes. MLR has been linked to poststroke depression (PSD) after three months and may have a role in the inflammatory process, according to previous research [17]. Cheng et al. discovered that a high MLR increased the risk of stroke-associated pneumonia (SAP), which can assist clinicians to diagnose patients with high-risk SAP earlier [18]. However, it is unclear if MLR has any clinical benefit in foretelling the emergence of AKI in acute ischemic stroke.

Given the potential role of inflammation in AIS and AKI, we proposed exploring the potential MLR role in AKI after AIS.

## 2. Material and Methods

### 2.1. Study Population

From January 2017 to December 2019, we included all patients (aged 18 or older) with AIS in the Department of Neurology Intensive Care Unit (NICU) at the University of South China's First Affiliated Hospital. The exclusion criteria included the following: ① presence of AKI before admission; ② preexisting chronic renal insufficiency or renal dysfunction requiring renal replacement therapy (RRT) before admission; ③ kidney transplantation; ④ admission to the NICU for less than 24 hours; ⑤ missing renal function or regular blood test results within two days of NICU admission; second AIS attack. Clinical symptoms and imaging exams were used to define AIS.

The primary outcome is the onset of AKI, while the secondary outcomes are in-hospital mortality, renal replacement therapy, ventilation, and hospital stay. This study, which was carried out in accordance with the Declaration of Helsinki, was approved by the First Affiliated Hospital of the University of South China.

### 2.2. Clinical Assessment and Data Collection

Sex, age, preexisting clinical conditions, complete blood count, blood biochemistry, inflammatory markers, and the National Institutes of Health Stroke Scale (NIHSS) score for neurological severity and Glasgow coma score (GCS) were all collected [19].

### 2.3. Acute Renal Damage Diagnosis

AKI was identified, according to the Kidney Damage: Enhancing Global Outcomes report (KDIGO) [20]. The minimum amount of serum creatinine found in the emergency hospital or general ward before admission to the NICU was used to calculate the benchmark creatinine level. When this value could not be acquired, the modifying of diet in renal disease (MDRD) approach was used to determine it, assuming a normal glomerular filtration rate of 75 ml · min^−1^ · 1.73 m^−2^ [21]. The estimated baseline glomerular filtration rate was calculated using the Chronic Kidney Disease Epidemiology Collaboration algorithm (eGFR) [22]. Utilizing the KDIGO recommendations, cases of AKI were identified: (a) a rise in serum creatinine (sCr) of 0.3 mg/dL (26.5 mol/l) within the previous 48 hours; (b) an increase in Scr to 1.5 times baseline within the previous 7 days; and (c) a urine flow rate of 0.5 ml/kg/h for six hours.

### 2.4. Neutrophil to Lymphocyte Ratio, Platelet to Lymphocyte Ratio, and Monocyte to Lymphocyte Ratio

The ratio of monocytes to lymphocytes was used to compute MLR, the ratio of neutrophils to lymphocytes was used to calculate NLR, and the ratio of platelets to lymphocytes was used to calculate PLR. MLR, NLR, and PLR were obtained from a standard blood test, and Roche's automatic analyzer identified the blood test as routine. MLR illustrates the interaction between innate (monocyte) and adaptive (lymphocyte) immune responses, including lymphocyte depletion and increase of monocytes.

### 2.5. Statistical Analysis

Continuous data showed variables as medians with interquartile ranges, while categorical data showed variables as frequency counts (percent). The chi-square test was used to compare groups in both categorical and continuous data. Spearman's correlation was used to look into how the MLR related to other variables. Using multivariable logistic regression, the MLR and NLR were examined for associations with the course of AKI and prognosis. The outcomes are shown as odds ratios (ORs). Using receiver-operating characteristic (ROC) curves, the predictive value of the MLR and NLR for the emergence of AKI and in-hospital mortality was assessed. Cut-off values, as well as the sensitivity and specificity for parameters, were computed using the Youden index. A two-tailed *p* < 0.05 indicated statistical significance for each study. The complete data analysis was carried out using SPSS 16. (Chicago, IL, USA).

## 3. Results

### 3.1. Basic Characteristics of Patients

**Figure 1:**
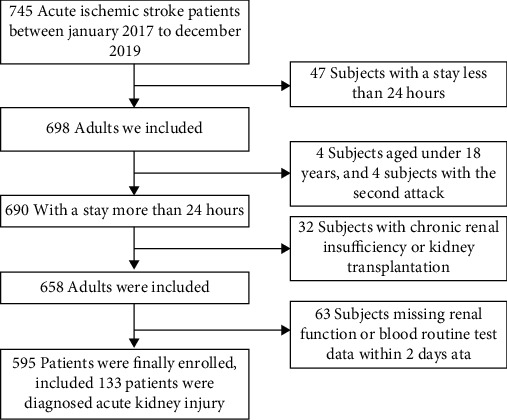
Flowchart of participant screening.

150 participants were eliminated from the research due to the baseline characteristics of the 745 patients who were screened for inclusion (Figure 1). In the end, 595 patients were recruited for the research analysis.

The baseline characteristics and results of the patients are summarised in Table 1. Participants included 361 (60.7%) men and 238 (39.3%) women, representing 66.27 ± 12.05 years of age. The eGFR was 76.58 ± 18.93, and the mean ± SD was 0.4729 ± 0.3461 for the MLR, 8.18 ± 5.45 for the NLR, and 192.46 ± 105.31 for the PLR. According to the KDIGO criteria, 133 patients (22.35%) were diagnosed with AKI, 104 were at stage 1, 24 were at stage 2, and 5 were at stage 3. There were remarkable differences flanked by the AKI set and nongroup in hypertension and diabetes, COPD, eGFR, baseline Scr, BUN, PCT, CRP, HGB, monocyte, MLR, NLR, and PLR. Additionally, individuals who possess AKI had a superior NIHSS and GCS than the non-AKI group. Moreover, patients who experienced AKI had a higher rate of CRRT and ventilation and a longer hospital stay. No significant differences were observed in age, coronary artery disease, albumin, triglycerides, white blood count, neutrophils, lymphocytes, or platelets between the two groups. The AKI group saw worse outcomes in comparison to the non-AKI group, including higher death rates and longer hospital stays (13.96 ± 12.86 vs. 11.70 ± 9.22, *p* = 0.024) and higher ventilation (12.8% vs. 5.6%, *p* = 0.005).

### 3.2. Association between MLR and Other Parameters

To investigate the relationship between the MLR and clinical factors, Spearman correlation coefficients were generated. Initial MLR was favourably correlated with CRP, PCT, NLR, PLR, NIHSS score, and monocytes at baseline (*p* < 0.05). In addition, age, triglycerides, ALB, HGB, lymphocytes, platelets, and GCS score were all linked negatively with baseline MLR (*p* < 0.05). However, there was no relationship between the baseline Scr and the MLR (Table 2).

### 3.3. By Logistic Regression, MLR and NLR for the Development of AKI

After correcting for sex, age, sepsis, hypertension, diabetes, COPD, baseline Scr, and eGFR, logistic regression was used to examine relationships between MLR and NLR with AKI after admission to the NICU. MLR and NLR were linked to the incidence of AKI in AIS patients, according to the analyses, with ORs of 3.652 and 1.163 (p0.001), respectively (Table 3). Meanwhile, the ORs of CRP and HGB for AKI incidence were 1.005 (1.00-1.010) and 0.986 (0.975, 0.997), respectively. However, PCT, triglycerides, and albumin levels also were not proposed as potential risk variables for AKI.

Age, sex, sepsis, hypertension, diabetes, coronary artery disease, COPD, Scr, and eGFR are all considered after adjusting for sex. Diabetes (DM) is denoted by the letters MLR (monocyte-to-lymphocyte ratio), CRP (c-reactive protein), PCT (procalcitonin), NLR (neutrophil-to-lymphocyte ratio), PLR (platelet-to-lymphocyte ratio), NIHSS (National Institutes of Health Stroke Scale score), and GCS (Glasgow Coma Score). ALB stands for albumin, HGB for haemoglobin, and Scr for serum creatinine. Diabetes is indicated by DM. The disorder that affects the lungs is called chronic obstructive pulmonary disease (COPD).

### 3.4. The ROC Curve of MLR and NLR with AKI Incidence

**Figure 2:**
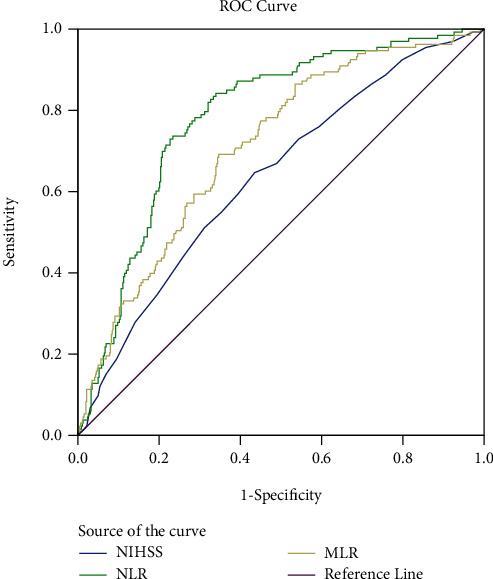
Receiver-operating characteristic curves for MLR, NLR, and NIHSS for predicting AKI. MLR: monocyte-to-lymphocyte ratio; NLR: neutrophil-to-lymphocyte ratio; PLR: platelet-to-lymphocyte ratio.

To assess the MLR and NLR's capacity for discrimination in the diagnosis of AKI, ROC curves were developed. AUCs for the MLR and NLR were 0.711. (95 percent CI: 0.663-0.758, *p* < 0.001) and 0.784 (95 percent CI: 0.742-0826, *p* < 0.001) for AKI prediction, respectively. MLR has the best ROC cut-off value of 0.4581, with 69.2% sensitivity and 65.4 percent specificity. The NLR has a cut-off value of 9.46 on the ROC curve, with 73.7 percent sensitivity and 76.6 percent specificity (Figure 2).

### 3.5. Multivariable Logistic Regression Predicts the Risk of In-Hospital Death

The in-hospital mortality-adjusted odds ratios based on MLR and NLR were 2.825 (1.058-7.545) and 1.085 (1.022, 1.151), respectively, after adjusting for sex, age, hypertension, COPD, or baseline Scr. In-hospital mortality risk for NIHSS was 1.199 (1.062, 1.354). There was no link between CRP and PCT and mortality at clinic (Table 4).

### 3.6. The ROC Analysis of MLR for In-Hospital Death

**Figure 3:**
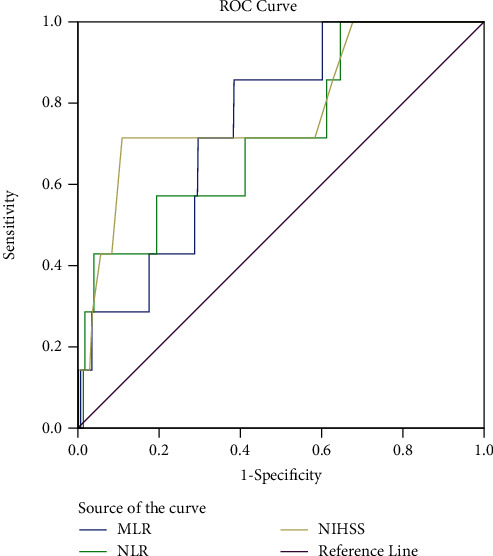
In-hospital mortality receiver-operating characteristic curves for MLR, NLR, and NIHSS. MLR stands for monocyte-to-lymphocyte ratio, while NLR stands for neutrophil-to-lymphocyte ratio, and PLR is for platelet-to-lymphocyte ratio.

With an ideal cut-off of 0.4731, 85.7 percent sensitivity, and 61.6 percent specificity, MLR produced an AUC of 0.745 (95 percent CI: 0.601-0.889, *p* = 0.026) for in-hospital mortality. For in-hospital mortality, NLR had an AUC of 0.724 (95 percent CI: 0.531-0.916, *p* = 0.042), with the optimal cut-off of 19.35, 57.1 percent sensitivity, and 96.1 percent specificity. With the optimal cut-off of 19.5, 71.4 percent sensitivity, and 89.89 percent specificity, 95 percent confidence interval for the AUC of NIHSS for in-hospital mortality was 0.587-0.976, *p* = 0.010 (Figure 3).

## 4. Discussion

The results of this study showed a relationship between the initial MLR and NLR evaluated at admission and the emergence of AKI in AIS patients. MLR and NLR both were higher in AKI patients than in non-AKI patients, with cutoff values of 0.4581 and 9.46, respectively. Furthermore, both the MLR and the NLR were found to be strongly linked to in-hospital death in patients admitted. NLR is an available, low-cost, and reproducible indicator of inflammation. It is also a marker of extensive secondary damage caused by neutrophils and their products. Therefore, NLR has been used to predict the prognosis of patients with sepsis with inconsistent results.

This is the first investigation examining the relationship between MLR and AKI in the setting of AIS. AKI was diagnosed in 133 (22.35 percent) of the patients in this study, which was higher than in a previous study [6]. This could be due to changes in the NICU patient demographic or the severity of the sickness. Male sex, hypertension, diabetes, and decreased eGFR have all been linked to an increased risk of acute kidney damage. An eGFR of less than 30 ml/min and having high blood pressure were identified to be risk factors for acute renal injury in AIS patients in a related study by Chusiri et al. [23].

Increasing evidence suggests that inflammatory and oxidative stress mechanisms initiated by inflammatory cells play a critical role in the pathological processes of ischemic stroke and its subtypes [24, 25]. Monocytes and activated macrophages can enter and adhere to the inner surface of the arterial wall by secreting various proinflammatory and prooxidant mediators [26].

Ischemia-reperfusion and inflammation may be key players in the aetiology of AKI, according to several experimental and clinical investigations [15, 27, 28]. The MLR has been hypothesised as a potential peripheral marker and an independent risk factor for ischemic stroke patients [29]. In this investigation, we discovered that in AIS patients, the MLR and NLR enhanced the likelihood of severe kidney damage. MLR and NLR may therefore be simple, practical, and affordable techniques in clinical practice for the prediction of AKI. There have been few studies on the MLR mechanism in AIS, and inflammation may be a crucial factor.

The AUC of the MLR for in-clinical death was 0.745 (95 percent CI: 0.601-0.889, *p* = 0.026), with the optimal cut-off value being 0.4731. In this study, we further examined the association between the MLR and NLR and in-hospital mortality. The 95 percent confidence interval for the AUC of the NLR for in-hospital mortality was 0.531-0.916, *p* = 0.042. A risk factor for hospital death is both MLR and NLR. The MLR and NLR also showed clear value for in-hospital mortality.

We also looked at RRT and ventilation as a secondary endpoint and discovered that there was a substantial difference in-hospital stay, ventilation, and RRT between AKI and non-AKI teams (*p* < 0.05), which was similar to Nadkarni et al.'s study [30].

The advantage of this study was to show that MLR and NLR were associated with a higher risk of AKI and in-hospital death in AIS patients. However, there are certain restrictions on this study that must be recognized. The first reason is that our investigation was a retrospective observational study carried out in a single facility. It was difficult to adjust for control variables and selective biases to prevent selection bias brought on by other factors; patients who spent less than 48 hours in the hospital or who had just one or two blood tests were excluded. As a result, the findings of this study can still be used to describe the epidemiological features of AKI in AIS patients. The diagnosis of AKI based on the Scr level and urine output, as well as histologic confirmation, was not accessible since diuretics might affect urine production, and histologic confirmation was not employed in the clinic. This could lead to an underestimation of the true occurrence of AKI. Finally, the study only looked at short-term prognoses; it neglected to look at long-term prognoses, which calls for long-term surveillance of AKI patients.

## 5. Conclusion

Our research supports the use of a single MLR and NLR measurement in the admission of AIS patients, and it may be a useful tool for the early detection of individuals who are at high risk of AKI. The MLR and NLR, meanwhile, help estimate in-hospital mortality.

## Figures and Tables

**Table 1:** Subjects' initial features and outcomes.

Variables	ALL (*n* = 595)	Patients with AKI (*n* = 133)	Patients without AKI (*n* = 462)	*p* value
Male (%)	361 (60.7%)	91 (68.4%)	270 (58.4%)	0.000
Age (years)	66.27 ± 12.05	68.17 ± 12.32	65.72 ± 11.93	0.823
Comorbidities				
Hypertension	436 (73.4%)	107 (80.5%)	329 (71.4%)	0.037^∗^
Diabetes	136 (22.9%)	43 (32.3%)	93 (20.2%)	0.003^∗^
Coronary artery disease	148 (24.9%)	41 (30.8%)	107 (23.2%)	0.071
COPD	13 (2.2%)	7 (5.3%)	6 (1.3%)	0.016
eGFR (ml/min/1.73m^2^)	76.58 ± 18.93	71.55 ± 22.82	78.03 ± 17.41	0.000
Laboratory index at NICU admission				
Baseline Scr (umol/l)	86.27 ± 35.76	99.21 ± 60.37	82.55 ± 23.29	0.000^∗^
BUN (mmol/l)	5.97 ± 3.65	8.11 ± 6.03	5.35 ± 2.24	0.000
Albumin (g/l)	40.14 ± 4.79	39.35 ± 5.59	40.36 ± 4.52	0.364
Triglyceride (mmol/l)	1.57 ± 1.42	1.38 ± 1.08	1.63 ± 1.50	0.087
PCT (ng/ml)	0.24 (0.07, 0.55)	0.54 (0.80, 0.72)	0.08 (0.05, 0.16)	0.001^∗^
White blood count/mm^3^	9.57 ± 3.77	10.61 ± 3.40	9.27 ± 3.66	0.314
CRP	11.29 (2.10,18.87)	25.78 (17.02,131.59)	8.47 (4.6, 56.08)	0.002^∗^
Haemoglobin	128.70 ± 20.68	124.49 ± 25.23	129.98 ± 19.01	0.005
NIHSS score	12.13 ± 6.61	14.51 ± 6.37	11.44 ± 6.52	<0.001^∗^
GCS score	11.26 ± 2.99	10.18 ± 3.18	11.57 ± 2.92	0.000
PLR	192.46 ± 105.31	230.71 ± 109.99	181.45 ± 101.40	<0.001^∗^
MLR	0.4729 ± 0.3461	0.6401 ± 0.4170	0.4248 ± 0.3069	<0.001^∗^
NLR	8.18 ± 5.45	11.65 ± 5.27	7.19 ± 5.08	<0.001^∗^
Neutrophils	7.77 ± 3.61	8.89 ± 3.82	7.44 ± 3.69	0.356
Lymphocytes	1.25 ± 0.63	1.12 ± 0.57	1.68 ± 0.63	0.092
Monocytes	0.49 ± 0.29	0.54 ± 0.33	0.48 ± 0.28	0.006
Platelets	201.91 ± 73.65	195.13 ± 72.47	203.87 ± 73.95	0.228
Outcome				
In-hospital mortality	11	4 (3.0%)	7 (1.5%)	0.026^∗^
RRT	2	2 (1.5%)	0	0.000^∗^
Ventilation	43 (7.23%)	17 (12.8%)	26 (5.6%)	0.005^∗^
Hospital stay	12.20 ± 10.12	13.96 ± 12.86	11.70 ± 9.22	0.024^∗^

MLR: monocyte-to-lymphocyte ratio; PLR: platelet-to-lymphocyte ratio; NIHSS: National Institutes of Health Stroke Scale score; GCS: Glasgow coma score; Scr: serum creatinine; RRT: renal replacement treatment; COPD: chronic obstructive pulmonary disease; CRP: C-reactive protein; PCT: procalcitonin; NLR: neutrophil-to-lymphocyte ratio; HGB: haemoglobin.

**Table 2:** The relationship between baseline MLR and other variables.

Variable	OR (95% CI)	*p* value
Age	-0.133	0.001^∗^
Baseline Scr (umol/l)	0.047	0.249
Triglyceride (mmol/l)	-0.085	0.045^∗^
CRP	0.297	<0.001^∗^
PCT (ng/ml)	0.120	0.008^∗^
ALB (g/l)	-0.091	0.026^∗^
NLR	0.562	<0.001^∗^
PLR	0.302	<0.001^∗^
HGB (g/dl)	-0.073	0.077
Neutrophils (count/mm^3^)	0.284	0.000^∗^
Lymphocytes (count/mm^3^)	-0.367	0.000^∗^
Platelets (count/mm^3^)	-0.05	0.021^∗^
NIHSS score	0.097	0.019^∗^
GCS score	-0.102	0.013^∗^
Monocytes (count/mm^3^)	0.575	0.000^∗^

MLR: monocyte-to-lymphocyte ratio; CRP: C-reactive protein; PCT: procalcitonin; NLR: neutrophil-to-lymphocyte ratio; PLR: platelet-to-lymphocyte ratio; NIHSS: National Institutes of Health Stroke Scale score; GCS: Glasgow coma score; HGB: haemoglobin; Scr: serum creatinine; ALB: albumin.

**Table 3:** values of the multivariable logistic regression analysis' MLR and NLR for AKI.

Variable	Unadjusted		Adjusted	
	OR (95% CI)	*p* value	OR (95% CI)	*p* value
MLR	6.135 (3.1797-11.772)	0.000	6.261 (3.142,12.477)	0.000
PCT (ng/ml)	1.005 (0.979-1.127)	0.174	1.035 (0.988-1.084)	0.151
CRP	1.006 (1.002-1.010)	0.005	1.005 (1.00-1.010)	0.036
WBC (count/mm^3^)	1.092 (1.040-1.146)	0.000	1.083 (1.028-1.140)	0.003
Albumin (g/l)	0.957 (0.920-0.996)	0.032	0.983 (0.951-1.026)	0.425
HGB (g/dl)	0.987 (0.978-0.996)	0.006	0.986 (0.975-0.997)	0.010
NLR	1.168 (1.119-1.218)	0.000	1.164 (1.114-1.215)	0.000
Triglyceride (mmol/l)	0.837 (0.682-1.029)	0.092	0.822 (0.621-1.021)	0.077
NIHSS	1.074 (1.042-1.108)	0.000	1.080 (1.045-1.115)	0.000
GCS	0.861 (0.809-0.918)	0.000		
Baseline Scr (umol/l)	1.012 (1.006-1.018)	0.000		
Sex	1.541 (1.023-2.321)	0.039		
Age (year)	1.018 (1.001-1.035)	0.040		
Sepsis	3.485 (0.217-56.091)	0.379		
Hypertension	1.651 (1.028, 2.652)	0.038		
DM	1.891 (1.231-2.902)	0.004		
Coronary artery disease	1.479 (0.965-2.266)	0.073		
COPD	4.222 (1.394-12.788)	0.011		

**Table 4:** The markers that predict in-hospital mortality.

Variable	Unadjusted		After adjustment	
	OR (95% CI)	*p* value	OR (95% CI)	*p* value
MLR	2.930 (1.118, 7.677)	0.029	2.825 (1.058, 7.545)	0.038
NLR	1.082 (1.021, 1.147)	0.008	1.085 (1.022, 1.151)	0.007
CRP	1.005 (0.993, 1.018)	0.397	1.005 (0.993, 1.018)	0.422
PCT	0.976 (0.672, 1.417)	0.898	0.979 (0.694, 1.382)	0.905
NIHSS	1.201 (1.065, 1.355)	0.003	1.199 (1.062, 1.354)	0.003
GCS	0.733 (0.584, 0.92)	0.007	0.728 (0.580, 0.914)	0.006

Adjusted by sex, hypertension, COPD, and Scr. MLR: monocyte-to-lymphocyte ratio; CRP: C-reactive protein; PCT: procalcitonin; NLR: neutrophil-to-lymphocyte ratio; PLR: platelet-to-lymphocyte ratio; NIHSS: National Institutes of Health Stroke Scale score; GCS: Glasgow coma score; HGB: haemoglobin.

## Data Availability

The raw data used to support the study's findings is given in the article's tables and figures.

## Abbreviations

AIS: Acute ischemic stroke
MLR: Monocyte-to-lymphocyte ratio
PLR: Platelet-to-lymphocyte ratio
NLR: Neutrophil-to-lymphocyte ratio
NICU: Neurology intensive care unit
PSD: Poststroke depression
AKI: Acute kidney injury
PCT: Procalcitonin
CRP: C-reactive protein
CystC: Cystatin C
Scr: Serum creation
WBC: White blood cell
HGB: Haemoglobin
COPD: Chronic obstructive pulmonary disease
DM: Diabetes mellitus
KDIGO: Kidney Disease: Improving Global Outcomes
KIM-1: Kidney injury molecule-1
ROC: Receiver-operating characteristic
BUN: Blood urea nitrogen
GFR: Glomerular filtration rate
NIHSS: National Institutes of Health Stroke Scale score
GCS: Glasgow coma score
AUC: Areas under the receiver-operating characteristic curve.
